# Effects of Postconditioning, Preconditioning and Perfusion of L-carnitine During Whole Period of Ischemia/ Reperfusion on Cardiac Hemodynamic Functions and Myocardial Infarction Size in Isolated Rat Heart

**Published:** 2013-04

**Authors:** Moslem Najafi

**Affiliations:** Department of Pharmacology and Toxicology, Faculty of Pharmacy, Tabriz University of Medical Sciences, Tabriz, Iran

**Keywords:** Hemodynamic, Ischemia, L-carnitine, Postconditioning, Preconditioning, Rat, Reperfusion

## Abstract

***Objective(s):*** In the present work, the effects of L-carnitine (LC) on postischemic cardiac hemodynamic functions and infarction size were studied in isolated rat heart.

***Materials and Methods:*** The hearts were subjected to 30 min regional ischemia followed by 120 min reperfusion. Then they were perfused by a drug-free or LC-enriched Krebs–Henseleit (K/H) solution during ischemia/ reperfusion (I/R) (Protocol 1), 10 min before ischemia induction (Protocol 2; preconditioning group) or the first 10 min of reperfusion (Protocol 3; postconditioning group).

***Results:*** The perfusion of LC in protocol 1 significantly reduced left ventricular end diastolic pressure (LVEDP) (*P<*0.05), and increased left ventricular developed pressure (LVDP) (*P<*0.05), rate pressure product (RPP) (*P<*0.01) and coronary flow rate (CFR) (*P<*0.05). The short-term preischemic administration of LC in protocol 2 improved RPP, CFR and decreased the extent of LVEDP elevation. However, protective effects of LC in this protocol were low compared to the whole period perfusion. In protocol 3, LC preserved postischemic cardiac functions not as much as the other protocols. In addition, infarct size significantly decreased by LC in all protocols as opposed to the control group (*P<*0.001).

***Conclusion: ***The results of the present work showed that LC produced protective effects against I/R injury. These protective actions were reversed by concomitant use of etomoxir (a CPT-I inhibitor), suggesting that the efficacy of LC could be due to its mitochondrial action, probably related to the raise in glucose oxidation of the reperfused hearts.

## Introduction

Carnitine is an essential cofactor under physiological conditions in the intermediary metabolism and transport of long-chain fatty acids (LCFAs) from the cytoplasm of cells to the mitochondrial matrix for ATP production (1-3). This pathway within the mitochondria is the major source of energy for the heart (4). Some previous studies have shown that L-carnitine (LC) exerts a protective effect against ischemia/reperfusion (I/R) injury, although controversial results are observed (1, 5-7). Arsenian *et al *(1996) demonstrated a decrease in mortality and the incidence of circulatory failure in a group of patients with acute myocardial infarction who were administered 3 g of LC along with the solution of glucose, insulin, potassium and magnesium (8). During ischemia, the accumulation of fatty acids and their intermediates has been shown to be deleterious to the recovery of the myocardial function of the reperfused heart (6). Some experimental and clinical studies have shown that LC reduces myocardial injury after I/R by counteracting the toxic effect of the high levels of free fatty acids, which occurs in ischemia (4, 7). In addition, LC reduces the intramitochondrial ratio of acetyl-CoA to free CoA, thus, it stimulates the activity of pyruvate dehydrogenase (PDH) and increases the oxidation of pyruvate (1, 9). It has also been shown that propionyl-L-carnitine, which penetrates faster than LC into myocytes, is effective in inhibiting the production of free radicals (10, 11). Other researchers and we previously reported the protective effects of LC on I/R- induced cardiac arrhythmias in isolated rat heart (8, 11, 12). In the study of Cui *et al* (2003), the effects of the short-term perfusion of LC on the incidence of reperfusion-induced arrhythmias and infarct size were investigated in isolated rat heart at the set of global ischemia. Results of this study showed that perfusion of LC for 10 min before induction of global ischemia failed to reduce the incidence of ventricular fibrillation. In addition, infarct size reduced only by high concentration (5 mM) of the agent (11). Previously, we reported protective effects of LC against reperfusion arrhythmias only when it is perfused for the whole period of 30 min ischemia and arrhythmogenic effects by short-term preischemic administration of the agent (13). 

Despite the mentioned protective effects of LC, its exact effects on postischemic cardiac hemodynamic functions and myocardial infarction size at the set of regional ischemia (not global ischemia) are not completely understood. Recently, we demonstrated that pharmacologically preconditioned rat hearts by LC had produced concentration-dependent recovery in cardiac functions and significant reduction in lactate accumulation (14). In the present work, the effects of LC on infarct size and postischemic cardiac hemodynamic functions including left ventricular end diastolic pressure (LVEDP), left ventricular developed pressure (LVDP), heart rate (HR), rate pressure product (RPP) and coronary flow rate (CFR) have been investigated in ischemic-reperfused isolated rat hearts in three different perfusion protocols. 

## Materials and Methods

*Chemicals*

The following chemicals were purchased: LC (Sigma Tau company), NaCl, NaHCO_3_, KCl, KH_2_PO_4_, MgSO_4_, CaCl_2_, D-glucose (Merck company), Sodium pentobarbital (Kela company, Belgium) and Heparin (Daru-pakhsh company, Iran).

*Animals*

Fifty male Sprague-Dawley rats (270-330 g) were used in this study. Animals were given food and water ad libitum. They were housed in the Animal House of Tabriz University of Medical Sciences at a controlled ambient temperature of 25±2°C with 50±10 % relative humidity and with a 12 hr light/12hr dark cycle (lights on at 7:00 a.m.). This study was performed in accordance with the Guide for the Care and Use of Laboratory Animals of Tabriz University of Medical Sciences (National Institutes of Health Publication No 85-23, revised 1985).

*Surgical procedure*

The animals were pretreated with intraperitoneal (IP) injection of 500 IU/kg heparin then anaesthetized by sodium pentobarbital (50-60 mg/kg, IP) (15). The rat hearts were excised rapidly and mounted on a non-recirculating Langendorff apparatus under 100 mmHg pressure at 37.5°C and perfused with modified Krebs–Henseleit (K/H) solution containing (in mM): NaCl (118.5), NaHCO_3_ (25.0), KCl (4.8), MgSO_4_ (1.2), KH_2_PO_4_ (1.2), D-glucose (12.0) and CaCl_2_ (1.7) that previously equilibrated with 95% O_2_–5% CO_2_. A fluid-filled balloon was introduced into the left ventricle and inflated to give a pre-load of 8–10 mmHg. An epicardial ECG was recorded during the experiment using two silver electrodes attached directly to the heart. Regional ischemia (30 min) was induced by the occlusion of left anterior descending coronary artery (LAD artery) and then by de-occluding of the coronary artery, the hearts were allowed to be reperfused (15).

*Measured parameters*

Hemodynamic factors including LVEDP, LVDP, RPP and HR were measured at regular intervals by Powerlab system (ADInstruments, Australia). CFR was measured by a time collection of the coronary perfusate that dripped from the heart. HR was calculated from the ECG. RPP was calculated by multiplying LVDP by HR (15).

*The determination of infarct size*

To determine infarct size, at the end of the 120 min reperfusion period, the ligature around the LAD artery was re-tied and the heart was slowly perfused with 2-3 ml of saline solution containing 0.25 % Evans blue dye (w/v) via the side arm of the aortic cannula. Perfusion of the hearts by Evans blue dye delineates the non-ischemic zone of the myocardium as a dark blue area. The hearts were frozen at -20°C and then the ventricles of the frozen hearts were sliced transversely in a plane perpendicular to the apico-basal axis into 2 mm-thick sections (14, 16). The slices were then incubated by 1 % (w/v) triphenyltetrazolium chloride (TTZ) solution in phosphate buffer (NaHPO_4_, 88 mM; NaH_2_PO_4_ 1.8 mM, pH= 7.4) for 15 min at 37°C to dye the non-infracted region (11, 17). This procedure resulted in the normally perfused tissue being stained blue, non-infracted, non-perfused tissue stained brick red and infarcted tissue remaining unstained and appeared pale. TTZ stains the non-infarcted myocardium a brick red color, indicating the presence of formazin precipitate that resulted from the reduction of TTZ by dehydrogenase enzymes present in viable tissue (16). The tissue slices were then fixed in 10% formalin for 24 h and then placed between two glass cover sheets. The sheets caused the tissue color to be clearly seen and also made a convenient flat surface for directly tracing the dimensions of the infarct and risk zone on a transparent sheet. The slices were drawn onto transparent sheets and by using a computerized planimetry package, the proportion of infracted tissue within the volume of myocardium at risk was calculated (16, 17).

*Experimental protocols*

Rats were allocated randomly to one of the following groups (n=6-8 in each group): (a) drug free control; (b) the hearts which were perfused with 0.5, 2.5 and 5 mM of LC-enriched K/H solution for the whole period of I/R (Protocol 1); (c) the hearts perfused with 0.5, 2.5 and 5 mM of LC-enriched K/H solution for 10 min before induction of ischemia (Protocol 2; preconditioning group); (d) the hearts perfused with 0.5, 2.5 and 5 mM of LC-enriched K/H solution during the first 10 min of reperfusion (Protocol 3; postconditioning group); (e) the infusion of 1 μM etomoxir (ETM; a carnitine palmitoyltransferase-I inhibitor) -enriched K/H solution in the absence or the presence (f) of LC (2.5 mM) for the whole period of I/R.

*Statistical analysis*

All results are expressed as Mean±SEM. A one-way ANOVA or repeated ANOVA with LSD post hoc test was used to test any difference between the groups for infarct size and hemodynamic factors, respectively. The differences between groups were considered significant at a level of *P<*0.05.

## Results

The effects of LC on cardiac hemodynamic functions during I/R

**Figure 1:**
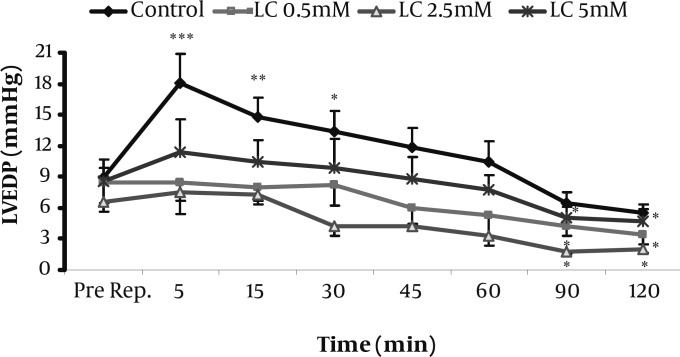
Effects of 0.5-5 mM L-carnitine on left ventricular end diastolic pressure (LVEDP) in the control and treated groups immediately before reperfusion and during 120 min reperfusion time (Protocol 1). Data are expressed as mean±sem. *** P<0.001, ** P<0.01, * P<0.05 versus pre-reperfusion value. (n=6-8 in each group). Pre Rep; Pre-reperfusion

The hemodynamic responses to the perfusion of 0.5, 2.5, and 5 mM of LC-enriched K/H solution for the whole period of I/R (Protocol 1) are summarized in Table 1. At the reperfusion phase, LVEDP was markedly increased in the control group especially during the first 30 min of reperfusion. However, as shown in Figure 1, all of the used concentrations of LC prevented this elevation and significantly reduced it during 90-120 min of reperfusion time (*P<*0.05 for all). In addition, LVDP, RPP and CFR were significantly increased by LC in this protocol at reperfusion phase (*P<*0.05, *P<*0.01 and *P<*0.05, respectively). In contrast to LC effects, co-administration of LC (2.5 mM) with 1 μM of etomoxir (ETM), increased LVEDP over most of the time steps of reperfusion (Table 2). The effect was significant during 5-30 min for both ETM and the co-administered group; however, the later group caused greater elevation in LVEDP at the same time. In contrast to LC, ETM and LC plus ETM did not show significant effects on LVDP and CFR at reperfusion phase while LC showed significant increase in LVDP especially during 5-30 min of reperfusion (Table 1).

**Table 1:** Effects of LC (0.5-5 mM) on LVEDP, LVDP, HR, RPP and CFR in the control and treated isolated rat hearts during pre-reperfusion and 120 min reperfusion in protocol 1. Data are expressed as Mean±SEM

Group	Factor	Pre-Rep	Reperfusion						
			5 min	15 min	30 min	45 min	60 min	90 min	120 min
Control	LVEDP	8.9±1.8	18.1±2.8***	14.7±2.0**	13.4±2.0*	11.9±1.8	10.4±2.0	6.5±1.0	5.5±0.9
	LVDP	100±16	117±16	115±13	108±10	104±14	100±10	90±12	80±13
	H R	224±15	240±23	224±16	219±17	244±17	220±17	227±16	190±13
	RPP	22319±1904	27697±2870	2522``4±1816	23384±1836	25525±2277	21250±1910	20619±2032	15054±1133*
	CFR	4.9±0.6	7.4±0.5*	6.6±0.5	5.6±0.5	5.1±0.5	4.6±0.5	4.2±0.5	3.9±0.7
LC (0.5 mM)	LVEDP	8.4±2	8.5±3.1	8.0±1.7	8.3±2.0	6.0±1.5	5.3±2.0	4.2±0.9*	3.4±0.9*
	LVDP	83±12	114±13*	117±8*	112±9*	100±10	95±9	89±14	82±10
	H R	214±21	263±14*	239±17	240±24	239±20	230±20	212±20	196±17
	RPP	17497±3141	29888±2639**	27857±1701**	26597±2205**	23978±2724*	22111±2341	19037±2376	16511±2185
	CFR	3.5±0.4	6.1±0.6*	5.6±0.5	5.2±0.5	4.8±0.5	4.2±0.5	4.0±0.3	3.5±0.3
LC (2.5 mM)	LVEDP	6.6±0.9	7.5±0.9	7.3±0.6	4.3±0.9	4.3±1.2	3.3±0.9	1.8±0.3*	2.0±0.4*
	LVDP	95±9	127±10*	122±9*	124±11*	112±12	114±11	97±11	83±9
	H R	223±25	251±27	259±23	243±21	245±26	243±21	238±19	232±15
	RPP	21205±2591	31601±3217**	31677±3186**	29347±3039**	27263±2923*	25321±3147	23291±2658	19119±1368
	CFR	3.9±0.4	6.6±0.4*	5.8±0.3	5.7±0.4	5.1±0.4	4.7±0.4	4.5±0.3	4.3±0.5
LC (5 mM)	LVEDP	8.6±1.3	11.4±3.1	10.5±2.0	9.9±2.8	8.8±2.2	7.8±1.3	5.1±1.0*	4.8±1.1*
	LVDP	92±9	139±16 **	133±9 **	120±9*	114±14	105±10	96±14	88±9
	H R	209±22	221±17	240±9	234±14	242±12	215±16	186±13	186±16
	RPP	18476±1251	31093±265***	31859±1060***	27974±1877**	26563±1113**	21506±1389	17583±1163	16558±1673
	CFR	4.6±0.7	7.1±0.9*	6.3±0.9	5.7±0.8	5.7±1.2	5.1±0.9	4.1±0.8	3.9±0.9

*** P<0.001, ** P<0.01,* P<0.05 versus pre-reperfusion value by using repeated ANOVA with LSD post test. n=6-8 in each group. LC; L-carnitine, LVEDP; left ventricular end diastolic pressure (mmHg), LVDP; left ventricular developed pressure (mmHg), HR; heart rate (beats/min), RPP; rate pressure product (mmHg.beats.min^-1^), CFR; coronary flow rate (ml/min), Pre-Rep; Pre-reperfusion time

**Figure 2:**
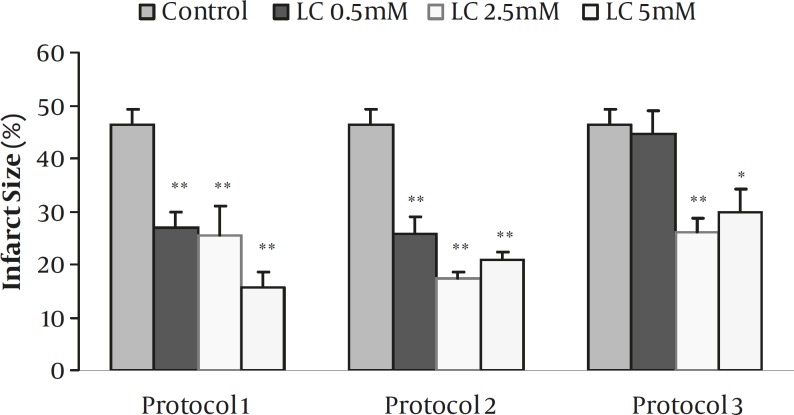
Myocardial infarct size as a percentage of risk zone volume in the control and isolated rat hearts receiving 0.5-5 mM L-carnitine (LC) during 30 min ischemia followed by 120 min reperfusion for the whole period of I/R (Protocol 1), for 10 min before induction of ischemia (Protocol 2) and during the first 10 min of reperfusion (Protocol 3). Data are expressed as mean±sem. ** *P*<0.001 and * *P*<0.01 versus control (n=6-8 in each group).

In protocol 2, the administration of LC by all used concentrations lowered the extent of LVEDP elevation and prevented LVDP reduction throughout the reperfusion time compared to the control group (Table 3). In addition, LC (2.5 and 5 mM) reserved RPP at the reperfusion time. Moreover, the improvement of RPP and LVDP by perfusion of 2.5 and 5 mM LC-enriched K/H solution also increased CFR throughout the reperfusion phase. The effect was significant during the first 30 min of reperfusion and 5 mM of LC showed a greater and longer effect. The results also showed that the administration of LC during the first 10 min of reperfusion (Protocol 3), by increasing LVDP, RPP and CFR caused some beneficial effects on cardiac function during reperfusion (Table 4). 

*Effects of LC on infarct size*

The effects of 0.5-5 mM of LC on infarct size for all protocols are summarized in Figure 2. In the control group, the infarct size was 46.3±2.9 % while the perfusion of 0.5, 2.5, and 5 mM of LC in protocol 1 reduced it to 27±2.9, 25.5±5.4 and 15.7±2.8 %, respectively (*P<*0.001 for all concentrations). Similarly, infarct size significantly decreased by administration of ETM (1 µM) alone or its co-administration with 2.5 mM LC (*P<*0.001, data not shown). Short-term perfusion of 0.5-5 mM LC 10 min before ischemia (Protocol 2) also produced significant reduction in infarct size by 25.9±3, 17.3±1.3 and 20.8±1.5 %, respectively (*P<*0.001 for all concentrations). Moreover, administration of 2.5 and 5 mM LC during the first 10 min of reperfusion (Protocol 3) also resulted in the reduction of infarct size (*P<*0.001 and *P<*0.01, respectively). However, 0.5 mM LC failed to reduce infarct size in this protocol. 

**Table 2:** Effects of ETM (1µM) with or without LC (2.5 mM) on LVEDP, LVDP, HR, RPP and CFR in the control and treated isolated rat hearts during pre-reperfusion and 120 min reperfusion in protocol 1. Data are expressed as Mean±SEM

Group	Factor	Pre-Rep	Reperfusion						
			5 min	15 min	30 min	45 min	60 min	90 min	120 min
ETM (1 µM)	LVEDP	9.3±1.4	13.0±1.6*	13.3±0.7*	14.0±0.6*	11.0±1.0	10.0±1.2	8.3±1.7	6.0±1.5
	LVDP	123±10	131±10	130±9	130±10	127±9	126±9	122±9	121±9
	H R	241±37	221±42	238±29	245±21	224±31	200±27	191±36*	166±39**
	RPP	29418±3949	28678±5081	30735±3261	31760±1973	28241±3398	24983±3059	23071±4081*	19923±4473**
	CFR	2.9±0.6	4.4±0.4	3.9±0.5	3.4±0.4	2.9±0.4	2.5±0.4	2.0±0.4	1.8±0.4
LC (2.5 mM) + ETM (1 µM)	LVEDP	12.4±1.9	17.5±4.3**	19.0±5.1**	20.0±4.8**	18.6±4.5**	16.0±3.7*	14.4±3.9	12.6±4.4
	LVDP	116±11	130±10	129±11	127±12	128±13	126±14	122±13	120±12
	H R	170±25	160±24	138±33	134±40	130±37	113±41*	107±53**	96±56 **
	RPP	16458±3083	20937±3498	18037±4679	17420±5644	19005±5194	14572±5313	13075±6440	11737±6888
	CFR	2.6±0.9	4.4±0.9	4.2±0.9	3.0±.0.9	2.6±0.9	2.2±0.9	1.7±0.8	1.5±0.7

** P<0.01, * P<0.05 versus pre-reperfusion value by using repeated ANOVA with LSD post test. n=6-8 in each group

**Table 3.** Effects of LC (0.5-5 mM) on LVEDP, LVDP, HR, RPP and CFR in the control and treated isolated rat hearts during pre-reperfusion and 120 min reperfusion in protocol 2. Data are expressed as Mean±SEM

Group	Factor	Pre-Rep	Reperfusion						
			5 min	15 min	30 min	45 min	60 min	90 min	120 min
Control	LVEDP	8.9±1.8	18.1±2.8***	14.7±2.0**	13.4±2.0*	11.9±1.8	10.4±2.0	6.5±1.0	5.5±0.9
	LVDP	100±16	117±16	115±13	108±10	104±14	100±10	90±12	80±13
	H R	224±15	240±23	224±16	219±17	244±17	220±17	227±16	190±13
	RPP	22319±1904	27697±2870	25224±1816	23384±1836	25525±2277	21250±1910	20619±2032	15054±1133*
	CFR	4.9±0.6	7.4±0.5*	6.6±0.5	5.6±0.5	5.1±0.5	4.6±0.5	4.2±0.5	3.9±0.7
LC (0.5 mM)	LVEDP	2.5±0.7	6.3±0.7*	7.3±2.3*	6.1±1.6*	5.3±1.9	4.5±1.4	3.5±0.9	3.0±0.9
	LVDP	112±12	125±9	121±9	117±8	114±10	111±16	112±9	110±8
	H R	177±16	184±42	201±22	185±23	179±21	179±15	140±20	114±15 **
	RPP	19880±1916	23027±5334	24260±2729	21619±2656	20445±2427	19873±1641	15472±2028	12340±1468**
	CFR	2.1±0.4	4.4±0.9	3.9±0.8	3.3±0.8	3.1±0.9	2.8±0.8	2.5±0.8	2.3±0.8
LC (2.5 mM)	LVEDP	4.5±0.7	9.2±2.4 *	8.5±0.9 *	8.0±1.0 *	5.8±0.8	4.8±1.2	3.5±1.2	3.3±1.4
	LVDP	116±14	131±12	136±15	132±14	130±16	127±15	119±15	115±19
	H R	198±16	227±17	212±10	205±10	216±15	221±13	189±21	178±13
	RPP	23328±2801	29856±2570*	29490±1339*	27578±1071	28264±3056	27997±2221	22410±2539	20362±1804
	CFR	3.3±0.3	6.1±0.5*	5.8±0.6*	5.1±0.5	5.0±0.5	4.6±0.5	4.0±0.5	3.3±0.4
LC (5 mM)	LVEDP	5.8±1.9	11.5±1.0**	10.0±2.0*	10.8±2.2*	9.5±1.0*	8.0±1.4	5.3±1.3	4.4±0.7
	LVDP	122±12	145±14	144±13	140±13	138±12	134±10	128±15	127±15
	H R	245±20	289±5	273±28	256±13	242±11	249±8	231±17	197±25
	RPP	30160±2736	41801±1367**	39496±5102**	35960±2463	33418±2341	33348±2493	29709±2713	24601±2996
	CFR	3.2±0.3	6.9±0.5**	5.9±0.4*	5.2±0.5*	4.5±0.4	4.2±0.6	3.7±0.5	3.5±0.5

*** P<0.001, ** P<0.01, *P<0.05 versus pre-reperfusion value by using repeated ANOVA with LSD post test. n=6-8 in each group. LC; L-carnitine, LVEDP; left ventricular end diastolic pressure (mmHg), LVDP; left ventricular developed pressure (mmHg), HR; heart rate (beats/min), RPP; rate pressure product (mmHg.beats.min^-1^), CFR; coronary flow rate (ml/min), Pre-Rep; Pre-reperfusion time

**Table 4:** Effects of LC (0.5-5 mM) on LVEDP, LVDP, HR, RPP and CFR in the control and treated isolated rat hearts during pre-reperfusion and 120 min reperfusion in protocol 3. Data are expressed as Mean±SEM

Group	Factor	Pre-Rep	Reperfusion						
			5 min	15 min	30 min	45 min	60 min	90 min	120 min
Control	LVEDP	8.9±1.8	18.1±2.8***	14.7±2.0**	13.4±2.0*	11.9±1.8	10.4±2.0	6.5±1.0	5.5±0.9
	LVDP	100±16	117±16	115±13	108±10	104±14	100±10	90±12	80±13
	H R	224±15	240±23	224±16	219±17	244±17	220±17	227±16	190±13
	RPP	22319±1904	27697±2870	25224±1816	23384±1836	25525±2277	21250±1910	20619±2032	15054±1133*
	CFR	4.9±0.6	7.4±0.5*	6.6±0.5	5.6±0.5	5.1±0.5	4.6±0.5	4.2±0.5	3.9±0.7
LC (0.5 mM)	LVEDP	4.4±0.7	7.2±1.8	6.4±1.6	5.6±1.1	7.0±3.2	5.3±2.3	3.7±1.7	3.7±1.1
	LVDP	102±16	124±13	120±10	116±11	117±12	109±14	96±17	92±17
	H R	219±22	212±43	225±23	229±11	211±9	208±10	178±26	158±18*
	RPP	22595±3431	26038±5197	27140±2552	26499±1396	24451±2027	22407±2478	16837±4264	14635±3507**
	CFR	4.4±0.7	6.6±0.6	6.5±0.8	5.7±0.8	5.3±1.0	4.8±1.0	4.3±1.0	4.0±1.1
LC (2.5 mM)	LVEDP	6.6±0.2	7.7±1.5	7.3±1.4	7.8±0.9	6.8±0.7	6.0±0.9	4.5±0.9	3.8±1.1
	LVDP	122±13	127±12	130±13	128±12	126±12	125±12	117±12	109±14
	H R	246±12	271±12	256±11	252±14	244±16	235±16	236±22	205±16
	RPP	30035±2069	34331±1954	32980±1420	31920±1371	30438±1510	29160±1745	27161±1727	21943±1273**
	CFR	6.2±1.9	8.8±0.7	7.5±0.6	6.3±0.8	5.2±0.7	4.4±0.7	3.8±0.7	3.2±0.4*
LC (5 mM)	LVEDP	6.2±1.3	13.5±1.5**	13.8±1.0**	10.4±0.4*	9.6±1.5	9.0±1.6	7.0±1.5	4.2±1.2
	LVDP	126±10	151±10 *	156±10 *	153±10 *	149±10	144±9	137±10	133±11
	H R	221±23	276±11*	236±26	254±12	252±8	251±7	241±11	233±12
	RPP	27894±3079	41489±2269***	36874±4168**	38686±1883**	37516±1411**	36138±1298**	33122±1463	33249±1721
	CFR	4.0±0.4	9.5±0.7***	7.4±0.6**	6.4±0.6	5.7±0.6	5.0±0.6	4.5±0.7	4.1±0.8

*** *P*<0.001, ** *P*<0.01, * *P*<0.05 versus pre-reperfusion value by using repeated ANOVA with LSD post test. n=6-8 in each group. LC; L-carnitine, LVEDP; left ventricular end diastolic pressure (mmHg), LVDP; left ventricular developed pressure (mmHg), HR; heart rate (beats/min), RPP; rate pressure product (mmHg.beats.min-1), CFR; coronary flow rate (ml/min), Pre-Rep; Pre-reperfusion time

## Discussion

The present study was focused on the pharmacological effects of LC on I/R- induced cardiac hemodynamic dysfunction and infarct size in isolated rat heart. The results showed that the perfusion of LC for the whole period of I/R (Protocol 1) significantly reduced LVEDP and increased LVDP, RPP and CFR at reperfusion phase. In contrast to LC alone, some beneficial effects of LC on cardiac hemodynamic functions were significantly reversed by ETM (a carnitine palmitoyltransferase-I inhibitor) as marked increase in LVEDP over most of the time steps of reperfusion (*P<*0.01). We suggested that ETM, through the inhibition of carnitine palmitoyltransferase-I, blocked LC, affects fatty acid metabolism in mitochondria. This effect might decrease ATP production and increase fatty acids and their toxic metabolites in the myocytes. The metabolites might probably result in some undesired alterations in cardiac functions after releasing during reperfusion. Yamada *et al *have shown that long-chain acylcarnitines accumulate in ischemic tissue and incorporate into cytosolic membrane compartments (18). Long-chain acylcarnitine esters are lipophylic and may readily damage membrane lipids and particularly, membrane bound enzymatic proteins (10), increase intracellular Ca^2+ ^(19), intracellular Na^+^ (20) and may thereby lead to electrophysiologic and contractile dysfunction in the myocardium (21). In addition, the accumulation of acylcarnitine esters in ischemic myocardium could contribute to the development of apoptosis (11). The short-term preischemic administration of 0.5-5 mM of LC in protocol 2 improved some cardiac functions as an improvement of RPP, CFR and lowering LVEDP elevation extent. However, protective effects of this protocol were low compared with protocol 1. Similarly, protocol 3 showed lower protective effects on hemodynamic factors in comparison with the other protocols. Based on the data, it seems that the administration of LC (0.5-5 mM) by inhibition of elevation in LVEDP, reducing LVEDP, and increasing LVDP followed by improving RPP (as a marker for heart performance) could recover hemodynamic functions of ischemic reperfused isolated rat hearts. 

Our findings also demonstrate that perfusion of LC (0.5-5 mM) causes marked and potent protective activity against I/R injuries as the reduction of infarct size in all protocols (*P<*0.001 for all concentrations). Profound reduction of infarct size by the short-term administration of LC (Protocols 2 and 3), suggests that the effects of LC on infarct size and hemodynamic functions is probably related to independent mechanisms. The results also suggest that the concentration and time of administration may play an important role in the pharmacologic effects of LC. Additionally, it seems that 2.5 mM of LC is the optimum concentration to improve cardiac hemodynamic functions and reduces myocardial infarct size in our model of study. The results of this work are consistent with the results of some previous studies (22, 23). It has been shown that in euglycemic and diabetic isolated rat hearts subjected to 20 min normothermic zero-flow global ischemia followed by 60 min reperfusion, LC (5 mM) preserves LVDP significantly without any significant difference between the groups regarding HR and CFR (24). In a similar study, normal and diabetic isolated rat hearts subjected to 60 min aerobic perfusion then 60 min low flow global ischemia and 60 min reperfusion, LC (10 mM) caused a significant increase in RPP because of improvement of LVDP (25). A part of the beneficial effects of LC on the recovery of cardiac function of diabetic and non-diabetic rat hearts may be mediated by its ability to stimulate glycolysis during the ischemia or glucose oxidation during aerobic reperfusion, or both (25). These experiments suggested that acute supplementation with LC significantly improved the tolerance of hearts from both diabetic and euglycemic rats to I/R interventions (24, 25). In carnitine deficient isolated rat hearts subjected to 20 min no flow ischemia followed by 30 min reperfusion, LC (5 mM) did not show significant effect on CFR (26). Previously, we demonstrated that LC pharmacologically precondition isolated rat hearts against ischemic and reperfusion injury in part by the recovery of postischemic ventricular hemodynamic functions, the depletion of glycogen and therefore reduction of lactate accumulation (14). In the study of Cui *et al *(2003), the perfusion of LC for 10 min before global ischemia improved the postischemic recovery of hemodynamic factors (CFR and LVDP) in the group perfused only with 5 mM (11). Although our results were consistent with the results of Cui *et al *in HR, CFR and LVDP, in contrast to their results, all the used concentrations of LC in our model significantly reduced infarct size, even the lowest concentration (0. 5 mM). We suggested that the existence of some methodological differences between the above studies (such as the type and duration of ischemia, the used concentrations of LC and experimental protocols) had an important role in those variations.

Clinically, oral carnitine therapy has been shown to be of clinical benefit for patients with coronary heart disease, heart and renal failure (7), cardiomyopathy, arrhythmias (2, 24) and acute myocardium infarction (22, 27). Tarantini *et al *(2006) reported prompt and subsequent maintenance administration of LC following an acute myocardial infarction inhibited progressive left ventricular dilatation (28). In small short-term studies, LC acts as an antianginal agent that reduces ST segment depression (1, 6) and LVEDP (6). Xue *et al* (2007) suggested that the beneficial effects of LC in cardiovascular disease were mainly related to restoration of energy saving and resumption of normal oxidative metabolism in the heart (23). Against I/R-induced injuries, the protective effects of LC can be related to different mechanisms such as stimulating fatty acid oxidation during ischemia, restoring the balance between fatty acid and glucose oxidation, reducing toxic effects of long chain free fatty acids metabolites (1, 10), mitigating the noxious effects of oxygen free radicals in the reperfused hearts (7, 10), increasing in coronary blood flow and anti-arrhythmic effect (10). Furthermore, researches suggest that carnitine is also crucial in the regulation of carbohydrate metabolism in addition to its role in the oxidation of fatty acids (1, 9). The cardioprotective action of LC against I/R injury was reversed by concomitant use of ETM in this study, suggesting that the efficacy of LC could be related to its mitochondrial action but it was not directly attributed to an increase in fatty acid oxidation. Instead, depending on the concentration and duration of administration, it appeared that LC might be beneficial in myocardial I/R by increasing PDH activity (1, 6) and shifting cellular energy production from oxidation of fatty acids to glucose (especially in isolated heart).

## Conclusion

In conclusion, our results showed cardioprotective effects of LC as the improvement of postischemic hemodynamic functions and reduced the extent of myocardial infarct size. The differences between the effectiveness of various protocols could be explained by the differences in duration and time of LC perfusion. Additionally, the results showed that 2.5 mM of LC was the optimum concentration to improve cardiac hemodynamic functions and protected isolated rat hearts against I/R-injury in our model of study. Further studies are required before carnitine administration could be recommended as a routine procedure in ischemic heart disease. 

## References

[1] Flanagan LJ, Simmons AP, Vehige J, Willcox DPM, Garrett Q (2010). Role of carnitine in disease. Nutr Metab.

[2] Oyanagi E, Yano H, Uchida M, Utsumi K, Sasaki J (2011). Protective action of l-carnitine on cardiac mitochondrial function and structure against fatty acid stress. Biochem Biophys Res Commun.

[3] Şıktar E, Ekinci D, Şıktar E, Beydemir S, Gülçin I, Günay M (2011). Protective role of L-carnitine supplementation against exhaustive exercise induced oxidative stress in rats. Eur J Pharmacol.

[4] Neely JR, Morgan HE (1974). Relationship between carbohydrate and lipid metabolism and the energy balance of heart muscle. Ann Rev Physiol.

[5] Arsenian MA (1997). Carnitine and its derivatives in cardiovascular disease. Prog Cardiovasc Dis.

[6] Ferrari R, Merli E, Cicchitelli G, Mele D, Fucili A, Ceconi C (2004). Therapeutic effects of L-carnitine on cardiovascular diseases. Ann NY Acad Sci.

[7] Calo AL, Pagnin E, Davis AP, Semplicini A, Nicolai R, Calvani M (2006). Antioxidant effect of l-carnitine and its short chain esters Relevance for the protection from oxidative stress related cardiovascular damage. Int J Cardiol.

[8] Arsenian MA, New PS, Cafasso CM (1996). Safety, tolerability, and efficacy of a glucose-insulin-potassium-magnesium-carnitine solution in acute myocardial infarction. Am J Cardiol.

[9] Lopaschuk G (2000). Regulation of carbohydrate metabolism in ischemia and reperfusion. Am Heart J.

[10] Lango R, Smolenski RT, Narkiewicz M, Suchorzewska J, Lysiak-Szydlowska W (2001). Influence of L-carnitine and its derivatives on myocardial metabolism and function in ischemic heart disease and during cardiopulmonary bypass. Cardiovasc Res.

[11] Cui J, Das DK, Bertelli A, Tosaki A (2003). Effects of L-carnitine and its derivatives on postischemic cardiac function, ventricular fibrillation and necrotic and apoptotic cardiomyocyte death in isolated rat hearts. Mol Cell Biochem.

[12] Najafi M, Garjani A (2005). The effect of L-carnitine on arrhythmias in the ischemic rat heart. Iran J Basic Med Sci.

[13] Najafi M, Garjani A, Maleki N, Eteraf Oskouei T (2008). Antiarrhythmic and Arrhythmogenic Effects of L-Carnitine in Ischemia and Reperfusion. Bull Exp Biol Med.

[14] Najafi M, Javidnia A, Ghorbani-Haghjo A, Mohammadi S, Garjani A (2010). Pharmacological Preconditioning with L-carnitine: Relevance to Myocardial Hemodynamic Function and Glycogen and Lactate Content. Pak J Pharm Sci.

[15] Najafi M, Ghaffary S, Shaseb E (2010). Effects of Acetyl-L-Carnitine on Cardiac Arrhythmias and Infarct Size in Ischemic-Reperfused Isolated Rat Heart. Iran J Basic Med Sci.

[16] Zacharowski K, Blackburn B, Thiemermann C (2001). Ranolazine, A partial fatty acid oxidation inhibitor, reduces myocardial infarct size and cardiac troponin T release in the rat. Eur J Pharmacol.

[17] Hausenloy JD, Duchen RM, Yellon MD (2003). Inhibiting mitochondrial permeability transition pore opening at reperfusion protects against ischemia–reperfusion injury. Cardiovasc Res.

[18] Yamada KA, Kanter EM, Newatia A (2000). Long-chain acylcarnitine induces Ca2+ efflux from the sarcoplasmic reticulum. J Cardiovasc Pharmacol.

[19] Clarke B, Wyatt KM, May GR, McCormack JG (1996). On the roles of long chain acyl carnitine accumulation and impaired glucose utilization in ischemic contracture development and tissue damage in the guinea-pig heart. J Mol Cell Cardiol.

[20] Wu J, Corr PB (1995). Palmitoylcarnitine increases [Na+]i and initiates transient inward current in adult ventricular myocytes. Am J Physiol..

[21] Haigney MCP, Lakatta EG, Stern MD, Silverman HS (1994). Sodium channel blockade reduces hypoxic sodium loading and sodium dependent calcium loading. Circulation.

[22] Singh RB, Niaz MA, Agarwal P, Beegum R, Rastogi SS, Sachan DS (1996). A randomised, double-blind, placebo-controlled trial of L-carnitine in suspected acute myocardial infarction. Postgrad Med J.

[23] Xue YZ, Wang LX, Liu HZ, Qi XW, Wang XH, Ren HZ (2007). L-carnitine as an adjunct therapy to percutaneous coronary intervention for non-ST elevation myocardial infarction. Cardiovasc Drugs Ther.

[24] Keller VA, Toporoff B, Raziano RM, Pigott JD, Mills NL (1998). Carnitine supplementation improves myocardial function in hearts from ischemic diabetic and euglycemic rats. Ann Thorac Surg.

[25] Broderick TL, Panagakis G, DiDomenico D, Gamble J, Lopaschuk GD, Shug AL (1995). L-carnitine improvement of cardiac function is associated with a stimulation in glucose but not fatty acid metabolism in carnitine-deficient hearts. Cardiovasc Res.

[26] Broderick TL, Cifuentes J, Green D, Paulson DJ (2001). Short-term carnitine deficiency does not alter aerobic rat heart function but depresses reperfusion recovery after ischemia. Can J Physiol Pharmacol.

[27] Colonna P, Iliceto S (2000). Myocardial infarction and left ventricular remodeling: results of the CEDIM trial, Carnitine Ecocardiografia Digitalizzata Infarto Miocardico. Am Heart J.

[28] Tarantini G, Scrutinio D, Bruzzi P, Boni L, Rizzon P, Iliceto S (2006). Metabolic treatment with L-carnitine in acute anterior ST segment elevation myocardial infarction. A randomized controlled trial. Cardiology.

